# Behavior of total alkaline phosphatase after radium-233 therapy in metastatic castration-resistant prostate cancer: a single-center, real-world retrospective study

**DOI:** 10.1590/0100-3984.2022.0080

**Published:** 2023

**Authors:** Flávia Paiva Proença Lobo Lopes, Marcos Villela Pedras, Álida Rosária Silva Ferreira, Patricia Massucheto Ledesma, Paulo Roberto Telles Pires Dias, Felipe Villela Pedras

**Affiliations:** 1 Clínica de Medicina Nuclear Villela Pedras, Rio de Janeiro, RJ, Brazil; 2 Universidade Federal de Minas Gerais (UFMG), Belo Horizonte, MG, Brazil; 3 Universidade Federal Fluminense (UFF), Niterói, RJ, Brazil

**Keywords:** Prostatic neoplasms, castration-resistant, Neoplasm metastasis, Alkaline phosphatase, Pragmatic clinical trials as topic, Radium/therapeutic use, Neoplasias de próstata resistentes a castração, Metástase neoplásica, Fosfatase alcalina, Ensaios clínicos pragmáticos como assunto, Rádio (elemento)/uso terapêutico

## Abstract

**Objective:**

To describe the behavior of total alkaline phosphatase (tALP) in patients with metastatic castration-resistant prostate cancer receiving radium-223 therapy, in a real-world scenario, and to describe overall survival (OS) among such patients.

**Materials and Methods:**

This was a retrospective study involving 97 patients treated between February 2017 and September 2020. Patients were stratified by the baseline tALP (normal/elevated). A tALP response was defined as a ≥ 30% reduction from baseline at week 12. For patients with elevated baseline tALP, we also evaluated treatment response as a ≥ 10% reduction in tALP after the first cycle of treatment. We defined OS as the time from the first treatment cycle to the date of death.

**Results:**

There was a significant reduction in the median tALP after each cycle of treatment (*p* < 0.05 for all). Data for tALP at week 12 were available for 71 of the 97 patients. Of those 71 patients, 26 (36.6%) responded. Elevated baseline tALP was observed in 47 patients, of whom 19 (40.4%) showed a response. Longer OS was observed in the patients with normal baseline tALP, in those with elevated baseline tALP that showed a response to treatment (≥ 10% reduction), and in those who received 5-6 cycles of therapy.

**Conclusion:**

The tALP may be used to predict which patients will benefit from treatment with a greater number of cycles of radium-223 therapy and will have longer OS.

## Abstract (pt)

**Objetivo:**

Descrever o comportamento da fosfatase alcalina total (tALP) em pacientes com carcinoma de próstata metastático resistente a castração, submetidos a terapia com rádio-223 em um cenário do mundo real, e a sobrevida global (SG) desses pacientes.

**Materiais e Métodos:**

Estudo retrospectivo envolvento 97 pacientes, no período de fevereiro/2017 a setembro/2020. Os pacientes foram estratificados de acordo com a tALP basal (normal/elevada). A resposta à tALP foi definida como uma redução em relação à linha de base de ≥ 30% na semana-12. Para pacientes com tALP basal elevada, também foi avaliada a resposta ao tratamento como uma redução de ≥ 10% de tALP após o primeiro ciclo. A SG foi definida como o tempo entre o primeiro ciclo e a data do óbito.

**Resultados:**

A redução da tALP média após cada ciclo foi significativa (*p* < 0,05). A tALP na semana 12 estava disponível para 71 dos 97 pacientes. Desses 71 pacientes, 26 (36,6%) responderam. Dezenove (40,4%) dos 47 pacientes com tALP elevada apresentaram resposta. Foi observada uma SG mais longa nos pacientes com tALP basal normal, nos pacientes com tALP basal elevada que apresentaram resposta ao tratamento (redução de ≥ 10%) e nos pacientes que receberam 5-6 ciclos.

**Conclusão:**

A tALP pode ser usada para prever parte dos pacientes que se beneficiarão do tratamento com um maior número de ciclos e uma SG mais longa.

## INTRODUCTION

Metastatic castration-resistant prostate cancer (mCRPC) is the most advanced stage of prostate cancer^(1)^ and occurs in 2-8% of all prostate cancer patients^(2)^. Up to 90% of patients with mCRPC have bone metastasis and approximately 80% have debilitating bone pain^(3)^.

Radium-223 is an alpha emitter that has been successfully used in men with mCRPC^(4-6)^, in whom it has been shown to increase overall survival (OS) and postpone skeletal-related events (SREs). It is a radionuclide that is a calcium analogue and therefore binds specifically to osteoblasts in the lesions. It has also been shown to inhibit the growth of bone metastases because it targets cancer cells, promoting a break in the double-stranded DNA, and acts in the metastatic bone microenvironment. However, its exact mechanism of action at the molecular level it is not yet fully understood^(7,8)^.

Because most studies of radium-223 therapy have been randomized clinical trials^(4,6,9-12)^, there is a lack of real-world evidence studies, especially in Brazil, that have evaluated radium-223 therapy regarding baseline patient characteristics and the best biomarker of treatment response. Real-world evidence studies utilize data on interventions used for health care decision-making, which are not collected as part of randomized controlled trials, and real-world evidence studies represent routine practice better than do randomized controlled trials, which are carried out under idealized conditions^(13)^. That makes them useful for validating the findings of clinical trials in real-world settings^(14)^. It is therefore meaningful to include real-world data in clinical decision-making.

The primary aim of this study was to describe the behavior of total alkaline phosphatase (tALP) in patients with mCRPC who have undergone radionuclide therapy with radium-223 in a real-world setting in Brazil. A secondary aim was to evaluate OS in that same population.

## MATERIALS AND METHODS

This was a real-world, single-center retrospective study, based on mCRPC patient records, designed to assess tALP behavior after radium-223 therapy. The baseline characteristics of the disease and the behavior of biomarkers are described. The study was approved by the Brazilian National Institutional Review Board (IRB; Reference no. 4.343.868; October 16, 2020). Because of the retrospective nature of the study, the IRB waived the requirement for informed consent.

All consecutive mCRPC patients treated with at least one cycle of radium-223 therapy, with a 4-week interval between cycles, between February 2017 and September 2020, were eligible for inclusion in the study. A total of 99 patients were invited to participate. Data collection began in October 2020 and concluded in April 2021. The patients were followed until loss to follow-up, death, or the end of February 2021 (the last follow-up evaluation). All data were managed, by the same physician, on Microsoft Excel. The duration of follow-up was calculated as the time in days from the first cycle of radium-223 therapy to the last recorded evaluation. Patients for whom there were missing tALP data (after the first cycle or at week 12) or for whom the date of the last follow-up evaluation was unknown were excluded from the specific analyses (tALP response and OS, respectively).

The following data were collected, when available, from patient records: age at diagnosis; age at the beginning of treatment; skin color; histological grade (Gleason score); Eastern Cooperative Oncology Group (ECOG) performance status^(15)^; numeric rating scale (NRS) pain score^(16)^; history of life-prolonging treatments for mCRPC performed before radium-223 therapy; levels of the biomarkers tALP, hemoglobin (Hb), prostate-specific antigen (PSA), and lactate dehydrogenase (LDH), before and after each cycle; number of cycles performed; and date of death (if applicable). The patients were stratified by the number of cycles of radium-223 therapy performed (one to six).

We evaluated treatment response considering tALP as a biomarker. The tALP after each cycle was compared with the baseline value (obtained before the initiation of treatment). For the sample as a whole, the tALP treatment response cutoff was initially defined as a ≥ 30% reduction from baseline at week 12. That value was adopted from the protocol established in a large randomized clinical trial of radium-223 therapy^(6)^. The patients were then stratified by the baseline tALP level-normal (≤ 130 U/L) and elevated (> 130 U/L)-in accordance with the host institution criteria, to identify possible differences between those with normal baseline tALP and those with elevated baseline tALP, in terms of the outcomes (treatment response and OS). In an additional exploratory analysis, restricted to patients with elevated baseline tALP, we defined treatment response as a ≥ 10% reduction from baseline, after the first cycle, following the protocol of a previous retrospective study^(17)^.

As a secondary endpoint, we calculated OS according to the number of cycles of radium-223 therapy performed, comparing the patients who underwent one to four cycles with those who underwent five or six cycles, as previously reported^(11,18)^. We defined OS as the time from the first radium-223 therapy cycle to the date of death, regardless of cause. We also attempted to determine whether baseline tALP would be a biomarker for longer OS. Other biomarkers (PSA, LDH, and Hb) and the ECOG performance status were also evaluated to determine whether they were associated with longer OS. Patients were stratified by the median values at baseline (above vs. below).

The ECOG performance status is scored as follows^(15)^: 0 = fully active; 1 = symptomatic but fully ambulatory; 2 = out of bed > 50% of the time; 3 = in bed > 50% of the time; and 4 = 100% bedridden. The NRS used here scores pain as follows^(16)^: 0 = no pain, analgesia not required; 1-3 = mild pain, non-narcotic analgesia required occasionally; 4-6 = moderate pain, interferes with daily activities; 7-10 = severe, disabling pain that leaves the individual unable to perform daily activities. The need for radiotherapy, the need for emergency skeletal surgery, and the presence of a pathological fracture were considered SREs if they occurred after the first radium cycle and within the first six months of follow-up. As previous life-prolonging systemic treatments, we considered chemotherapy and androgen-receptor targeting therapy.

In the descriptive analysis, categorical variables are presented as absolute and relative frequencies. Continuous variables are presented as median and interquartile range (IQR), because the Shapiro-Wilk test showed that they had a nonparametric distribution. In the analysis of biomarker behavior, we used t-tests for paired samples to compare the medians. For the OS analysis, we used chi-square tests to compare groups and the Kaplan-Meier estimate of survival probability to compare survival times between groups. Significance tests for survival time and hazard ratios were performed using the log-rank test. Values of *p* < 0.05 were considered significant. All analyses were processed in the program R, 2021^(19)^.

## RESULTS

**Figure 1:**
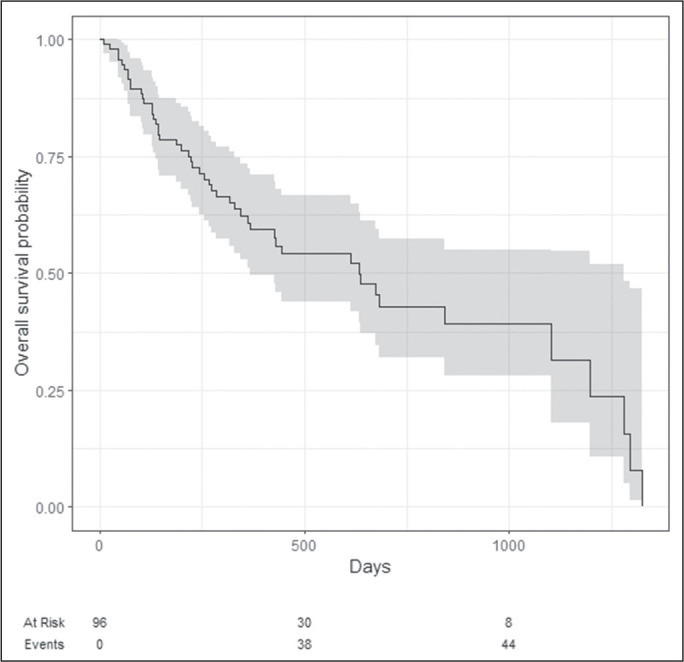
Chart of OS in the sample as a whole, with confidence intervals (gray shading), numbers of patients at risk, and numbers of events.

Of the 99 patients with mCRPC evaluated initially, two (2.0%) were excluded during data verification. The baseline clinical characteristics of the study sample are shown in Table 1. The median age at diagnosis was 64 years (IQR, 41-91 years), and the median age at the beginning of treatment was 74 years (IQR, 48-93 years). The median follow-up period (after the last cycle of treatment) was 11 months (IQR, 1-45 months). Of the 97 patients, 88 (90.7%) self-reported their skin color as white. Prostatectomy was performed in 47 patients (48.5%). As illustrated in Figure 1 (with 95% confidence intervals), the median OS was 634 days.

**Table 1:** Clinical and demographic characteristics of patients with mCRPC receiving radium-223 therapy.

Characteristic	(N = 97)
Gleason score, n (%)	
6	12 (12.4)
7	17 (17.5)
8	25 (25.8)
9	22 (22.7)
10	7 (7.2)
No data	14 (14.4)
Age (years) at diagnosis, median (IQR)	64 (41-91)
Age (years) at the beginning of radium-223 therapy, median (IQR)	74 (48-93)
Life-prolonging treatments before radium-223 therapy, n (%)	
None	3 (3.1)
One	29 (29.9)
Two	37 (38.1)
Three	28 (28.9)
ECOG performance status**^(15)^**, n (%)	
0 (fully active)	13 (13.4)
1 (symptomatic but ambulatory)	51 (52.6)
2 (out of bed < 50% of the time)	23 (23.7)
3 (in bed > 50% of the time)	9 (9.3)
4 (100% bedridden)	1 (1.0)
Baseline tALP^*^, n (%)	
≤ 130 U/L	42 (47.2)
> 130 U/L	47 (52.8)
Pretreatment NRS pain score**^(16)^**, n (%)	
0 (no pain)	6 (6.2)
1-3 (mild pain)	30 (30.9)
4-6 (moderate pain)	27 (27.8)
7-10 (severe pain)	34 (35.1)
Number of completed cycles of treatment, n (%)	
1	8 (8.2)
2	9 (9.3)
3	10 (10.3)
4	9 (9.3)
5	5 (5.2)
6	56 (57.7)

* Values available for only 89 patients.

In most of the patients, the Gleason score was 8 (in 25.8%) or 9 (in 22.7%), indicative of poorly differentiated tumors; the baseline ECOG performance status was 1 (in 52.6%) or 2 (in 23.7%); and there was moderate or severe pain before the initiation of treatment (in 62.9%). Previous life-prolonging therapies consisted of hormone therapies (in 82.5%) and chemotherapy (in 48.5%). Most of the patients (57.7%) completed the treatment (six cycles of radium-223 therapy). Only three patients (3.1%) received radium-223 therapy as the first-line treatment, whereas 37 (38.14%) received it as a third-line treatment. Eight patients (9.3%) experienced an SRE after the initiation of radium-223 therapy: intractable bone pain/rapid lesion progression in five; pathologic bone fracture (without concomitant administration of a bone protector) in two; and spinal cord compression in one.

**Figure 2:**
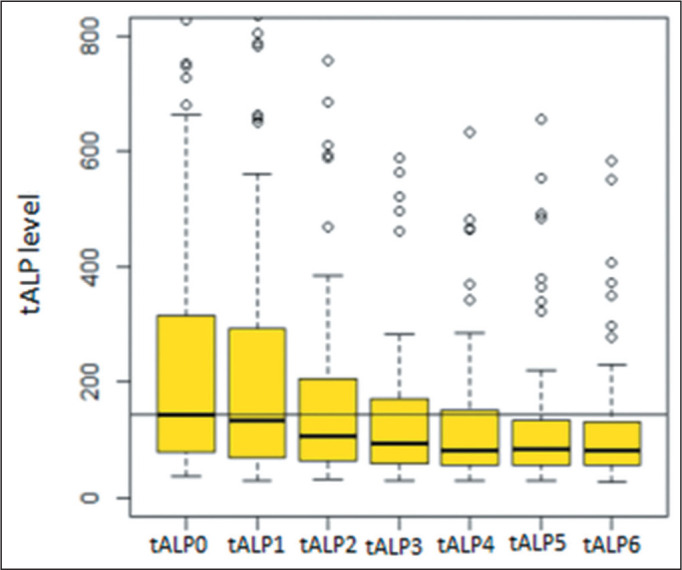
Box plot of tALP behavior during radium-223 therapy.

Baseline tALP data were available for only 89 patients, among whom the median value was 143 U/L (IQR, 36-1421 U/L). Of those 89 patients, 42 (47.2%) had a normal baseline tALP (≤ 130 U/L-median, 70 U/L; IQR, 36-125 U/L) and 47 (52.8%) had an elevated baseline tALP (> 130 U/L-median, 291 U/L; IQR, 135-1421 U/L). As can be seen in the box plot (Figure 2), there were significant reductions in the median tALP after each cycle, except after the first cycle, the data for which were not included in the analysis.

Black line, median at baseline; tALP0, baseline tALP; tALP1, tALP after the first cycle; tALP2, tALP after the second cycle; tALP3, tALP after the third cycle; tALP4, tALP after the fourth cycle; tALP5, tALP after the fifth cycle; tALP6, tALP after the sixth cycle.

When considering baseline tALP, regardless of treatment response, we found that OS was longer in the patients with normal baseline tALP than in those with elevated baseline tALP (hazard ratio, 0.30; 95% CI: 0.15-0.57; *p* < 0.001). For the analysis of treatment response at week 12 (≥ 30% reduction in tALP from baseline), data were available for 71 patients. Of those, 26 (36.6%) showed a response. However, the difference in OS between the responders and nonresponders, regardless of the baseline tALP, was not significant (*p* = 0.4). Among the responders, there was also no statistical difference in OS between those with normal baseline tALP and those with elevated baseline tALP. However, when the tALP cutoff point for treatment response was a ≥ 10% reduction from baseline, 19 (40.4%) of the 47 patients with elevated baseline tALP showed a treatment response and the OS was longer for the responders (hazard ratio, 0.48; 95% CI: 0.23-0.99; *p* = 0.04). Other baseline characteristics that showed a significant association with longer OS were an ECOG performance status of 0 or 1 (*p* < 0.001); an NRS pain score of 0-2 (*p* = 0.008); baseline Hb > 11.9 (*p* = 0.003); and below-normal baseline LDH (*p* = 0.01). In contrast, OS was significantly longer among the patients with above-normal baseline PSA (*p* = 0.003). Longer OS was also found to be significantly associated with having received more than four cycles of radium-223 therapy (*p* < 0.001).

In assessing the number of completed cycles, we observed an increase in the number of cycles (five or six), per patient, over the years. In 2020, 84.6% of patients with mCRPC completed five or six cycles, compared with only 25.6% in 2017. That improvement in the number of cycles performed is probably attributable to patients now being referred to the clinic at an earlier stage of the disease and the use of better inclusion criteria for treatment.

## DISCUSSION

In bone metastases, osteogenesis occurs through bone resorption and the formation of new bone tissue, resulting in a vicious and unbalanced cycle^(3,5)^. Therefore, patients can experience bone pain and SREs, such as pathological fractures and spinal cord compression, as well as potentially requiring radiation therapy or palliative bone surgery^(3,5,7)^. For patients with bone metastases, one of the aims of the available treatments, like radium-223 therapy, is to try to prevent SREs and prolong OS^(7)^. One of the great advantages of radium-223 therapy is that it has the capacity to bind directly to such metastases^(5,7)^.

Bone metastasis in prostate cancer is mainly osteoblastic and can be detected through imaging (bone scintigraphy or positron emission tomography/computed tomography with fluoride) or through blood and urine assays. In alkaline pH, the catalyst enzyme ALP promotes hydrolysis of phosphate monoesters and is well established as an important marker that indirectly quantifies osteoblastic activity. It is found in various tissues, such as bone, liver, intestinal, renal, testicular, and placental tissues. The measurement of tALP considers all of those isoforms, in which bone-specific ALP (BSAP) is included^(20)^. Together, BSAP and nonspecific hepatic ALP account for approximately 90% of the tALP, therefore being the two most abundant isoforms^(21)^.

Because tALP and BSAP can both be evaluated on a routine basis, they have been used, separately or together, as biomarkers of bone formation. Both have been used in order to predict bone turnover in prostate cancer since 1936^(22)^. Although tALP is widely used in clinical practice, some studies have demonstrated that BSAP has greater sensitivity as a biomarker of bone turnover. The use of tALP and BSAP together has been considered the gold standard, especially for the study of new potential biomarkers for prognostic evaluation in patients with prostate cancer, such as P1NP, which is a metabolite directly associated with bone formation^(23)^. In the present study, tALP was used as a biomarker because it is used on a routine basis at the host institution to monitor the treatment response to radium-223 therapy, as has been done in other studies^(4,6,9,11)^.

Although we observed a significant reduction in the median tALP after each cycle, only 36.6% of the patients showed a ≥ 30% reduction from baseline at week 12. However, in a randomized, double-blind, placebo-controlled study of radium-223 therapy applying that same criterion^(4)^, 47.0% of the patients showed a treatment response. That difference might be explained by the lower number of patients or by the possibility that patients with higher baseline tALP are less likely to present a reduction in tALP during treatment, as has been observed in other studies^(24)^.

In our sample of patients with mCRPC, the median baseline tALP was 143 U/L, which is considered high. Similar findings were reported by van der Doelen et al.^(24)^, who reported a median baseline tALP of 156 U/L in their sample of 180 patients with mCRPC. Those authors defined treatment response as a ≥ 10% reduction in tALP from baseline after the first cycle of radium-223 therapy and thus identified a treatment response in 62% of the patients. When we applied the same criterion to our sample, the treatment response rate was 40.4%. The difference in response rate between our two studies might be due to smaller size of our sample.

Some of the baseline characteristics known to be potential keys to radium-233 treatment success are PSA, ECOG performance status, pain status, baseline tALP, and Hb^(9,18,25)^. Frantellizzi et al.^(26)^ found no differences between responders and nonresponders in terms of age and baseline characteristics, although they found that a three-variable score could be a useful predictor of treatment success. They showed that patients with an ECOG performance status of 0 or 1, PSA < 20 ng/mL, and Hb > 12 g/dL were likely to show a better response. In the present study, longer OS was significantly associated with an ECOG performance status of 0 or 1, an NRS pain score of 0-2, a baseline Hb > 11.9, and below-normal LDH. However, in our sample, OS was significantly longer among the patients with higher PSA.

Regardless of other characteristics, a greater number of cycles of treatment is consider one of the main predictors of prolonged OS^(18,25-28)^. In the present study, OS was significantly longer among the patients receiving five or six cycles of radium-223 therapy than among those receiving fewer cycles.

Our study has several limitations. First, the retrospective design precludes any inferences regarding causality. Another limitation is the fact that we did not evaluate other potential biomarkers that might predict a better treatment response. In addition, we had limited access to genetic data and some laboratory test results were missing. Furthermore, the small sample size limits the generalizability of our results. Nevertheless, we have described the experience of a single center in a real-world scenario, and the results obtained are similar to those of comparable multicenter studies. We have also shown that the learning curve is a reality.

Prospective studies are still needed in order to establish a multifactorial score to predict treatment response in patients with mCRPC^(29)^. In addition, there is a need for real-world studies in Brazil to determine how to obtain the most benefit from radium-223 therapy and make it possible for patients to subsequently receive as many other modalities as possible in order to increase OS. Therefore, appropriate treatment planning can offer patients the best treatment approach in each phase of the disease, according to their characteristics.

In conclusion, tALP behavior may be used as a biomarker of a response to radium-223 therapy and of prognosis in patients with mCRPC, allowing clinicians to identify those who will benefit from the treatment. It is possible that the cutoff for treatment response should be lower for patients with a higher baseline tALP, although further studies are needed in order to test that hypothesis. It also appears that a greater number of cycles of treatment (specifically five or six cycles) prolongs OS, regardless of other characteristics.
